# Long-term optical coherence tomography changes and visual outcomes after vitrectomy for epiretinal proliferation: lamellar holes and full-thickness macular holes

**DOI:** 10.1186/s40942-025-00711-3

**Published:** 2025-07-24

**Authors:** Tarin T. Tanji, Jae Young Heo, Terrence Murphy, Vivek Chaturvedi

**Affiliations:** 1 Department of Ophthalmology, Rush University Medical Center, Chicago, IL 60612 USA; 2 Department of Ophthalmology, The Ohio State University Wexner Medical Center, Columbus, OH 43210 USA; 3 Illinois Retina Associates, Chicago, IL 60657 USA

**Keywords:** Macular hole, Pars plana vitrectomy, Optical coherence tomography, Full-thickness macular hole, Lamellar hole

## Abstract

**Background:**

Epiretinal proliferation (ERP) is an extension of preretinal tissue, creating a thin, semi-translucent layer of fibrous tissue on the retina. ERP has been recently associated with degenerative lamellar macular holes (DLMH) and full-thickness macular holes (FTMH). The proposed surgery for patients has been vitrectomy and ERM peeling, but there is no consensus on whether DLMH is a stable condition or should be resolved with surgical treatment. The purpose was to investigate optical coherence tomography (OCT) changes and visual outcomes of degenerative LMH and FTMH secondary to ERP after pars plana vitrectomy (PPV) and internal limiting membrane (ILM) peeling.

**Methods:**

This retrospective case series evaluated 14 eyes with DLMH and eight eyes with FTMH. All 22 eyes were associated with ERP and treated with PPV with ILM peeling. Best-corrected visual acuity (BCVA, in logMAR), OCT findings, including presence of foveal bump and ellipsoid zone integrity, were documented. Wilcoxon signed-rank tests were used for numerical data. Statistical significance was based on a value of *p* < 0.05.

**Results:**

Among the 14 DLMH cases, the mean Snellen equivalent VA was 20/75 at baseline; 20/59 at the 4–12-month postoperative period; and 20/56 at the final follow-up visit. For the eight FTMH cases, the mean Snellen equivalent VA was 20/94 at baseline; 20/45 at the 4–12-month postoperative period; and 20/38 at the final follow-up visit. Compared to baseline VA, FTMH cases demonstrated a statistically significant improvement in the 4–12-month postoperative VA (*p* < 0.05) and in the final postoperative VA (*p* < 0.05). Among all cases, a strong correlation was observed between baseline logMAR and change in logMAR from baseline to final follow-up (R^2^ = 0.671).

**Conclusion:**

This study demonstrated that surgical intervention led to improved visual and anatomic outcomes in patients with DLMH and FTMH, with a correlation between baseline VA and postoperative visual outcomes. The results suggest that patients with worse preoperative VA experienced the greatest benefit in visual outcome from PPV and ILM peeling. Future studies would look at the timing of when to ideally intervene to improve anatomic and visual outcomes.

## Introduction

Epiretinal proliferation (ERP) is an extension of preretinal tissue, creating a thin, semi-translucent layer of fibrous tissue found on the inner surface of the retina [1]. ERP was first discovered by Witkin et al. in 2006 as a thick membrane observed on optical coherence tomography (OCT) [2]. This preretinal tissue has been recently associated with degenerative lamellar macular holes (DLMH) and full-thickness macular holes (FTMH) [1, 3]. 

A lamellar macular hole (LMH) is a vitreoretinal disorder characterized by a partial thickness macular defect, an irregular foveal contour, splitting of the inner and outer retinal layers, and an intact photoreceptor layer [4, 5]. The prevalence of LMHs is 1.1 to 3.6% of the population, with no significant correlation with age [4, 6]. LMH was first proposed by J. D. Gass in 1975 when identifying a macular lesion resulting from cystoid macular edema [7]. Subsequently, the advancement of OCT has greatly enhanced the capability to assess and diagnose LMH. Additional parameters are now considered when describing LMH. These holes can exhibit varying levels of disruption to the photoreceptor structure, potentially affecting the ellipsoid layer, and they may sometimes also be linked with the presence of an epiretinal membrane (ERM) [8]. 

**Fig. 1:**
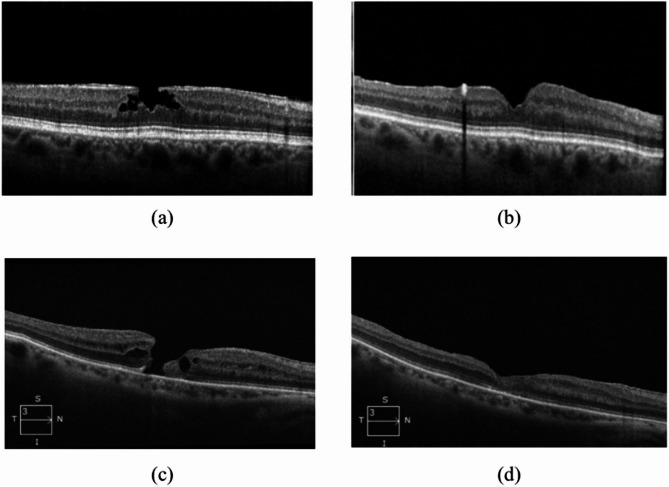
Example of a degenerative lamellar macular hole (DLMH) at baseline with a visual acuity (VA) of 20/40 (**a**) and at the final postoperative visit, 24 months later, with VA of 20/60 (**b**). Example of a full-thickness macular hole (FTMH) at baseline with a VA of 20/80 (**c**) and at the final postoperative visit, three months later, with VA of 20/80 (**d**)

LMHs are further classified into degenerative lamellar macular holes (DLMHs) and tractional lamellar macular holes (TLMHs) [9]. TLMH is a partial-thickness macular lesion characterized by an intact ellipsoid layer along with cystic changes, often associated with tractional ERM. In contrast, a DLMH typically presents with intraretinal cavitation, an ellipsoid defect, and lamellar hole epiretinal proliferation (LHEP) [9, 10]. Representative OCT images of a DLMH and FTMH in this study are shown in Fig. 1.

Previous case series have shown mixed visual outcomes after pars plana vitrectomy (PPV) with membrane peel for DLMHs [3, 9–14]. The proposed surgery for patients has been vitrectomy and ERM peeling, especially for patients with TLMH, but there is no consensus on whether DLMH is a stable condition or should be resolved with surgical treatment [8, 11]. FTMH traditionally occurs as a result of vitreomacular traction; however, it can also occur without any vitreomacular traction and in the presence of ERP [3]. Another challenge with DLMH and FTMH secondary to ERP is the uncertain natural history and the timing of when a retinal specialist should aim to intervene surgically. The aim of this study was to investigate the surgical intervention of vitrectomy and membrane peel in a large case series of ERP macular pathology, which included both DLMH and FTMH, with the goal of adding to the literature. We hypothesize that surgical intervention for DLMHs results in better visual outcomes. To our knowledge, this descriptive study presents one of the largest series of consecutive surgical cases of DLMH.

## Methods

This retrospective chart review evaluated 22 eyes with a history of PPV for ERP maculopathy (14 DLMH and eight FTMH). Patients were examined over a five-year period, from January 2015 to December 2020. Data were queried using the Current Procedural Terminology code 67,041 from Rush University Medical Center (Chicago, IL, USA) and Illinois Retina Associates, an associated private retina practice (Chicago, IL, USA). The Rush University Medical Center Institutional Review Board and Ethics Committee determined that approval was not required for this study because it satisfied the exemption criteria of involving the collection of data and documents for research and was recorded in an anonymous manner such that subjects cannot be identified directly or through identifiers linked to the subject. This study was conducted according to the principles of the Declaration of Helsinki, regulations, and guidelines. No financial support was obtained in the completion of this study.

OCT was performed using Cirrus HD-OCT (Carl Zeiss Meditec). OCT images of the macula were analyzed for all patients with DLMH by a retinal specialist (V.C.). OCT images were taken before and after the surgical intervention of vitrectomy and membrane peel. The OCT criteria for diagnosing DLMH followed the criteria from the OCT-based consensus definition.^15^ Included cases had undergone surgery with a follow-up duration of at least three months. Exclusion criteria were the presence of macular degeneration, branch retinal vein occlusion, diabetic retinopathy, any other maculopathy, previous macular laser, history of cystoid macular edema, vitreomacular traction, and previous retinal surgery.

Baseline data included the patient’s sex, age, baseline Snellen visual acuity (VA), and indication for surgical procedure. OCT imaging data included presence of foveal bump and ellipsoid zone (EZ) defect. Intraoperative data included the type of tamponade used. Postoperative data included the presence of EZ defect and any changes in EZ defect at the final follow-up appointment. Snellen VA values were converted to Logarithm of the Minimum Angle of Resolution acuities (logMAR) for data analysis. Improvement in VA was defined as an increase of two or more lines on the Snellen VA chart from baseline, stable vision was within one line above or below the baseline VA, and decrease in VA was a reduction of two or more lines from baseline.

Microsoft Excel was used to collect de-identified patient data. The statistical analysis was performed using SPSS^®^ Statistics (IBM Corp., Armonk, NY, USA) software. Wilcoxon signed-rank tests were used for numerical data (logMAR values). Statistical significance was based on a value of *p* < 0.05.

### Surgical procedure

Vitrectomy was performed using 23- or 25-gauge cannulas, placed 3.4–5 mm to the limbus, in a standard fashion by ten surgeons. ERP and ILM peeling was performed on all patients with indocyanine green or tissue blue staining and peeled with forceps and/or membrane scrapers. Foveal ILM sparing was not performed in any of the surgeries. A gas bubble was placed in 19 eyes (86.4%), including air (*n* = 3), 20–25% sulfur hexafluoride (SF_6_) (*n* = 13), or 14% perfluoropropane (C_3_F_8_) (*n* = 3). All surgeries were performed at Illinois Retina Associates (Chicago, IL, USA).

## Results

### Baseline data

The study population included 22 eyes in 22 patients, including 14 eyes (63.6%) with DLMH and eight eyes (36.4%) with FTMH (Table 1). The mean patient age was 70.1 ± 7.8 years (range: 55–81 years). The mean patient follow-up was 46.7 ± 28.2 months (range: three to 101 months). Preoperatively, foveal bump was found in 31.8% (7/22 cases). Among the 14 DLMH eyes, EZ disruption was found in 12 eyes (85.7%). The average duration of ERP recorded at our practice was 11.4 months (range: six days to 73 months). At baseline, 12 eyes (54.5%) were pseudophakic, and at the final exam, 22 eyes (100%) were pseudophakic.

**Table 1:** Baseline patient characteristics

Characteristics	Sample Size (*n* = 22)
Indication for Surgery	
Degenerative lamellar macular hole, n (%)	14 (63.6)
Full-thickness macular hole, n (%)	8 (36.4)
Age Group (years; mean ± SD)	70.1 ± 7.8
Mean follow-up duration, mos ± SD	46.7 ± 28.2
Gender	
Female, n (%)	13 (59.1)
Male, n (%)	9 (40.9)
Right eyes, n (%)	9 (40.9)
Pseudophakic, n (%)	12 (54.5)
Pseudophakic by final exam, n (%)	22 (100)
Epiretinal proliferation, n (%)	22 (100)
Foveal bump, n (%)	7 (31.8)

*SD* Standard Deviation

### Change in visual acuity

Among the 14 DLMH cases, the mean VA was 0.57 logMAR (20/57 Snellen equivalent [SE]) at baseline, 0.47 logMAR (20/59 [SE]) at the 4–12-month postoperative period, and 0.44 logMAR (20/56 [SE]) at the final follow-up appointment (Table 2). In the 4–12-month postoperative period, vision had improved in six eyes (42.9%), was stable in seven eyes (50.0%), and had decreased in one eye (7.1%). At the final postoperative visit, vision had improved in four eyes (28.6%), was stable in nine eyes (64.3%), and had decreased in one eye (7.1%).

**Fig. 2:**
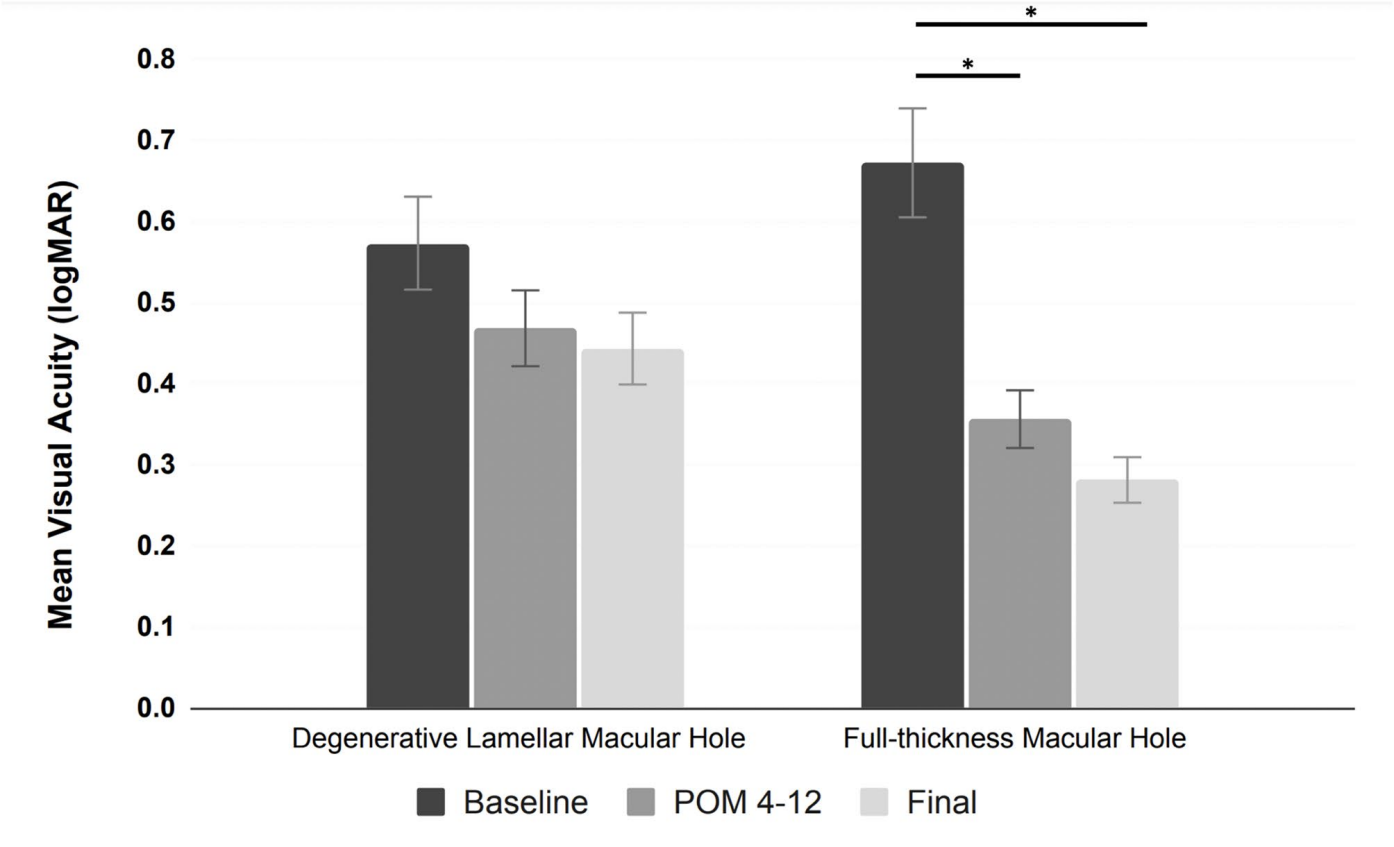
Preoperative and postoperative Snellen visual acuity for patients who had vitrectomy for degenerative lamellar macular hole or full-thickness macular hole. The first bar in each group is the preoperative VA; the second bar is the postoperative month 4–12 VA; and the third bar is the final VA. Asterisks denote a statistically significant difference in VA.

Among the eight FTMH cases, the mean VA was 0.67 logMAR (20/94 [SE]) at baseline; 0.36 logMAR (20/45 [SE]) at the 4–12-month postoperative period, and 0.28 logMAR (20/38 [SE]) at the final follow-up appointment. In the 4–12-month postoperative period, vision had improved in six eyes (75.0%), was stable in two eyes (25%), and had decreased in none. At the final postoperative visit, vision had improved in six eyes (75.0%), was stable in two eyes (25.0%), and had decreased in none. A statistically significant improvement was observed at 4–12-month postoperatively (*p* < 0.05) and in the final postoperative VA (*p* < 0.05) (Fig. 2).

**Fig. 3:**
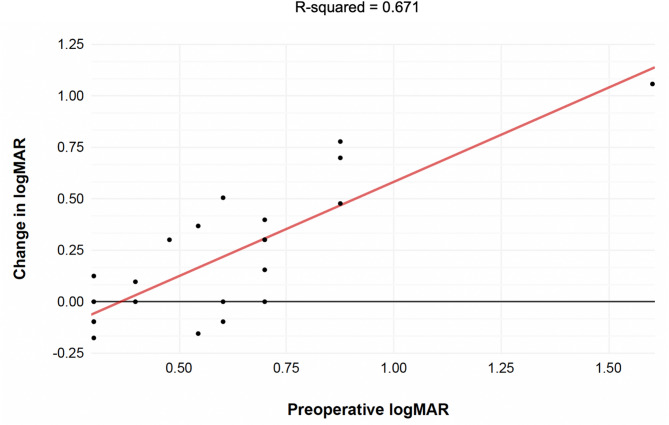
Univariate regression analysis using the preoperative logMAR as a predictor for positive change in logMAR (baseline to final appointment)

There was a correlation between the change in logMAR and preoperative logMAR (R^2^ = 0.671) (Fig. 3). Those with worse preoperative VA tended to have the largest improvement in VA at their final postoperative visits.

**Table 2:** Mean BCVA before and after PPV

	Preoperative	4–12 month postoperative	Final
DLMH			
logMAR	0.57	0.47	0.44
Snellen	20/75	20/59	20/56
* p* value	-	0.15	0.19
FTMH			
logMAR	0.67	0.36	0.28
Snellen	20/94	20/45	20/38
*p* value	-	< 0.05	< 0.05

*BCVA* Best Corrected Visual Acuity, *PPV* Pars Plana Vitrectomy, *DLMH* Degenerative Lamellar Macular Hole, *FTMH* Full-Thickness Macular Hole

### Anatomic characteristics

There were two postoperative complications after PPV. One patient with a DLMH developed hypotony, which self resolved within one week. This patient’s Snellen VA improved from baseline 20/60 to a final VA of 20/30. Another patient developed a macular hole following PPV for DLMH, and a second PPV was performed six weeks later. This patient’s Snellen VA improved from baseline 20/40 to 20/30 at the last postoperative visit with an anatomically closed macular hole on spectral domain OCT.

Among the 14 DLMH cases, 12 cases had a preoperative EZ junction disruption. At the final postoperative visit, EZ junction disruption was restored in seven eyes (58.3%). In this group, the mean baseline VA improved from 20/96 (logMAR 0.68) to 20/55 (logMAR 0.44) at final follow-up. Among the remaining five eyes, EZ junction was still present but improved in four eyes (33.3%) and still disrupted in one eye (8.3%). In this group, the mean baseline VA improved from 20/60 (logMAR 0.48) to 20/50 (logMAR 0.3944) at final follow-up. However, the improvement in VA, as measured in logMAR, was not statistically significant (*p* = 0.62).

## Discussion

Since Gass first discovered LMH in 1975, two distinct subtypes of LMH have been identified: tractional and degenerative [9, 15]. Tractional is characterized by presence of ERM with tractional signs, vitreomacular traction, intraretinal schisis, with a mustache appearance, while the degenerative type is characterized by intraretinal cavitation involving all layers of the retina, frequent presence of LHEP, and a top-hat appearance [9, 13, 15]. 

Furthermore, advancement of OCT technology has enabled recognition of two types of epiretinal material: LHEP and standard ERM [13]. LMHs with LHEP have distinct characteristics from LMHs with standard ERM alone. Previous studies have shown LMH with LHEP have a poorer BCVA, thinner central foveal thickness (CFT), and disruption of outer retinal bands, compared to LMH with standard ERM alone [1, 9, 15–18]. 

Previous studies have demonstrated mixed visual outcomes of surgical intervention for DLMHs, with some studies suggesting avoiding peeling DLMHs due to worse postoperative vision and the absence of tractional membranes [11, 17–21]. More recently, with newer techniques, such as the ERP embedded technique, VA improvement has been demonstrated [22]. Our study demonstrated improvement in vision after PPV and ILM peeling without ERP embedded technique. Compared to baseline VA, there was a statistically significant improvement in the 4–12-month postoperative VA (*p* < 0.01) and in the final postoperative VA (*p* < 0.01). At the final visit, vision had improved or remained stable in 95.4% (21/22 eyes). Among all 22 eyes, the mean baseline VA was 20/81 and mean final VA was 20/48 (*p* < 0.01). Our visual outcomes were consistent with a case series by Figueroa et al., who demonstrated that BCVA improved in DLMH (*p* < 0.012) [13]. Furthermore, a meta-analysis by Parisi et al. showed a significant visual improvement in the DLMH and LHEP groups (*p* < 0.001) [17]. 

FTMH can also develop without vitreomacular traction and in the presence of ERP [3]. The FTMH group did have a more statistically significant improvement in final VA compared to the DLMH eyes. This is likely secondary to the different foveal pathology in each condition. The central VH loss can tend to be more profound in a FTMH given the total loss of all retinal layers in the fovea (with restoration postoperatively), compared to a DLMH where there is significant variability in the foveal integrity of the EZ.

The EZ status (disrupted vs. intact) is considered to reflect photoreceptor function, and is often used as an indicator of visual outcomes in many retinal diseases [23]. Similarly, our findings also show that those eyes with restored EZ status had improved VA at the final follow-up visit. In our DLMH cohort, seven out of 12 eyes had baseline EZ disruption that had resolution at the final follow-up visit. In this group, the mean baseline VA improved from 20/96 to 20/55 at final follow-up. The remaining five eyes, which either showed improvement or no change in EZ disruption, demonstrated a mean VA improvement from 20/60 to 20/50 at final follow-up. Of note, some eyes can show an improvement, but not necessarily a fully restored EZ.

Previous studies have reported an incidence of postoperative FTMH of up to 30% [11–13, 24, 25]. Of the 22 eyes in this study, one developed FTMH after surgery that was successfully closed on the second procedure. It has been advocated to peel the ILM with sparing of the fovea to reduce the risk of FTMH development postoperatively [10, 22, 26]. Our study included multiple surgeons, none of which employed this technique.

A strength of this study is that all cases were performed by retinal surgeons who used a similar surgical technique. Limitations of this study include its small sample size, retrospective design, and lack of a comparator arm group of patients DLMHs who did not undergo surgery. Due to limitations in patient history, we could only track the duration of ERP prior to surgery in our practice at Illinois Retina Associates.

Further inquiry as to the timing of when we should intervene for these DLMH would be beneficial. The timing of ERM removal has been a more recent interest in the literature, including attention from the Diabetic Retinopathy Clinical Research Network Protocol AM. Another future research consideration is continuing to further understand the natural history of DLMHs, which could help direct retinal specialists when to intervene to avoid permanent retinal damage.

This study demonstrated that surgical intervention led to improved visual and anatomic outcomes in patients with DLMH and FTMH, with a correlation between baseline VA and postoperative visual outcomes. The results suggest that patients with worse preoperative VA experienced the greatest benefit in visual outcome from PPV and ILM peeling.

## Data Availability

The data that support the findings of this study are not openly available due to reasons of sensitivity and are available from the corresponding author upon reasonable request.
